# An Improved Dynamic Model for the Respiratory Response to Exercise

**DOI:** 10.3389/fphys.2018.00069

**Published:** 2018-02-07

**Authors:** Leidy Y. Serna, Miguel A. Mañanas, Alher M. Hernández, Roberto A. Rabinovich

**Affiliations:** ^1^Biomedical Engineering Research Centre (CREB), Automatic Control Department, ESAII, Universitat Politècnica de Catalunya, Barcelona, Spain; ^2^Biomedical Research Networking Center in Bioengineering, Biomaterials and Nanomedicine (CIBER-BBN), Madrid, Spain; ^3^Bioinstrumentation and Clinical Engineering Research Group - GIBIC, Bioengineering Department, Engineering Faculty, Universidad de Antioquia (UdeA), Medellín, Colombia; ^4^ELEGI and COLT Laboratories, Centre for Inflammation Research, Queen's Medical Research Institute, University of Edinburgh, Edinburgh, United Kingdom; ^5^Department of Respiratory Medicine, Royal Infirmary of Edinburgh, Edinburgh, United Kingdom

**Keywords:** respiratory system, dynamic modeling, exercise simulation, work of breathing, respiratory control, computational modeling

## Abstract

Respiratory system modeling has been extensively studied in steady-state conditions to simulate sleep disorders, to predict its behavior under ventilatory diseases or stimuli and to simulate its interaction with mechanical ventilation. Nevertheless, the studies focused on the instantaneous response are limited, which restricts its application in clinical practice. The aim of this study is double: firstly, to analyze both dynamic and static responses of two known respiratory models under exercise stimuli by using an incremental exercise stimulus sequence (to analyze the model responses when step inputs are applied) and experimental data (to assess prediction capability of each model). Secondly, to propose changes in the models' structures to improve their transient and stationary responses. The versatility of the resulting model vs. the other two is shown according to the ability to simulate ventilatory stimuli, like exercise, with a proper regulation of the arterial blood gases, suitable constant times and a better adjustment to experimental data. The proposed model adjusts the breathing pattern every respiratory cycle using an optimization criterion based on minimization of work of breathing through regulation of respiratory frequency.

## Introduction

The respiratory system is a complex and feedback system which is responsible for supplying sufficient oxygen (*O*_2_) for metabolism and eliminating carbon dioxide (*CO*_2_) produced by metabolic reactions in order to keep the homeostasis of arterial blood gases and pH in any situation and, particularly, during exercise (Duffin, 2013). For achieving this goal, the respiratory control system regulates pulmonary ventilation so that at equilibrium gas exchange in the lungs matches metabolism: *O*_2_ provision and *CO*_2_ elimination in the lungs equal *O*_2_ consumption and *CO*_2_ production in the tissues (Bell, 2006; Duffin, 2013; Guyton, 2015).

The regulation of *O*_2_ is achieved keeping a partial pressure of *O*_2_ (*P*_*O*_2__), in the arterial blood, that saturates arterial hemoglobin and provides a sufficient gradient to supply the tissue metabolic. Because of the hemoglobin can be saturated within a wide range of *P*_*O*_2__, oxygen is mainly regulated when its saturation falls to hypoxia limits (*P*_*O*_2__ < 70 mmHg) (Roussos and Koutsoukou, 2003; Duffin, 2013). On the other hand, due to *CO*_2_ diffuses more easily than *O*_2_ and quickly reacts with *H*_2_*O*, generating hydrogen ion concentrations ([*H*^+^]), the regulation of *CO*_2_ is more difficult to achieve although it can carry out by controlling the partial pressure of *CO*_2_ (*P*_*CO*_2__) and, therefore, [*H*^+^] (Duffin, 2013).

Pulmonary ventilation (${\dot{V}}_{E}$) is normally generated through controlled contraction and relaxation of respiratory muscles during inspiration and expiration, respectively. The pressures generated by them combined with the airway flow resistance, and the lung elastance determine the airflow and lung volume, correspondingly (Bianchi et al., 1995). For this, the respiratory control system, which is responsible for automatic control of breathing, (a) receives and integrates several afferent inputs from both central and peripheral chemoreceptors and pulmonary sensors, to determine ventilatory demand and efficiently adjust tidal volume (*V*_*T*_) and respiratory frequency (*f*_*R*_); and (b) provides signals to the phrenic and intercostal moto-neurons, which drive the diaphragm and intercostal muscles (Duffin, 1994; Bianchi et al., 1995). Due to this, the respiratory control system is frequently seen as a pattern central generator that seeks to maintain healthy levels of *O*_2_, *CO*_2_, and pH in body and brain tissues (Feldman et al., 2013; Richter and Smith, 2014). This control can also be affected by behavioral inputs such as speech, voluntary control of respiratory muscles and wakefulness state (Duffin, 2013).

The central chemoreceptors, which are located in the medulla, sense increases of [*H*^+^] in their local environment and, therefore, increases in the brain partial pressure of *CO*_2_ (*P*_*bCO*_2__) (Nattie and Li, 2009). On the other hand, peripheral chemoreceptors, which are located in the carotid bodies, sense changes in the arterial partial pressures of *CO*_2_ and *O*_2_ (*P*_*aCO*_2__ and *P*_*aO*_2__, respectively) (Marshall, 1994). In this way, both the central and the peripheral chemoreceptors allow matching pulmonary ventilation to tissue metabolism via a chemical arc that includes the respiratory control system, the chemoreceptors and respiratory muscles (Duffin, 2013). Recently, changes in *P*_*aO*_2__ have been related to increases of chemoreceptor sensitivity to [*H*^+^] at extreme hypoxia (Blain et al., 2010; Duffin, 2013).

During mild and moderate exercise, metabolic rate and pulmonary exchange increase as a result of muscular activity, such increases quickly produce a higher ventilation which seeks to prevent hypercapnia (due to increase in *CO*_2_ production) and hypoxia (due to increase in *O*_2_ consumption) (Turner et al., 1997; Whipp and Ward, 1998; Haouzi, 2006). Because of ${\dot{V}}_{E}$ increases nearly immediately at the onset of exercise, levels of *CO*_2_ and *O*_2_ remain practically unchanged from their rest values so, for this reason, a respiratory drive is not compatible with sensing an error signal transported in the blood (Duffin, 1994).

In recent decades, several clinical and experimental investigations have been carried out to determine the control mechanisms responsible for adjusting ventilation during exercise. A challenging aspect of this ventilatory stimulus is that ${\dot{V}}_{E}$ increases while the brain and arterial partial pressures of *CO*_2_ and *O*_2_ remain almost unchanged. A generally accepted theory is the so-called neurohumoral theory (Turner, 1991; Mateika and Duffin, 1995; Turner et al., 1997; Whipp and Ward, 1998), which is mainly based on the respiratory system response to a step workload. In this theory, respiratory dynamic response from rest or light to moderate exercise is characterized by three phases: the first one, determined by a sudden increase of ${\dot{V}}_{E}$, the second one, by a gradual and exponential increase of ${\dot{V}}_{E}$ and the third one, by its value in the steady state. The “abrupt” increase of ${\dot{V}}_{E}$ is usually attributed to neurogenic mechanisms, since this increase is considered too fast to be explained by humoral agents, such as central and peripheral chemoreceptors, due to delayed transport. Until now, such mechanisms are not yet well-understood, because they seem to involve feed-forward control systems or learned processes that have not been clearly figured out (Bell, 2006; Williamson, 2010).

Some studies establish that the behavior of ventilatory response during moderate exercise is related to the frequency of limb movement, and the force carried out by exercising muscles (Duffin, 1994). On the other hand, other studies establish that such response is mainly based on factors related to gas exchange more than factors related to the motor activity (Haouzi, 2006). A broad review on the mechanisms currently implicated in the control of breathing at the onset of exercise from a perspective of an integrated system can be found in Bell (2006) and Duffin (2014).

Many empirical and functional models have been proposed in the literature to describe numerous aspects of the respiratory system (Fincham and Tehrani, 1983; Butera et al., 1999a,b; Cheng et al., 2010; Williamson, 2010; Tsai and Lee, 2011; Cheng and Khoo, 2012; Serna Higuita et al., 2014; Serna et al., 2016; Diekman et al., 2017). Due to the primary goal of this system is to regulate the *CO*_2_ and *O*_2_ in the brain and body tissues, ventilatory stimuli like exercise, hypoxia and hypercapnia are frequently used to evaluate the performance of such models. Exercise has been one of the most used ventilatory stimuli for validating this kind of models and their control mechanisms (Magosso and Ursino, 2005; Hermand et al., 2016).

Particularly, Hermand et al. (2016) have presented a mathematical model that allows analyzing the mechanisms responsible for the instability of the respiratory control system under simultaneous metabolic (exercise), and environmental (hypoxia) stresses. In this case, the model analysis is mainly focused on variations of ${\dot{V}}_{E}$ and *f*_*R*_ taking into account several settings to simulate the central and peripheral chemoreceptor responses. On the other hand, Maggoso and Ursino have presented a respiratory model that allows obtaining the transient and steady-state cardiorespiratory response to exercise with a good performance, but it does not include a comprehensive cardiovascular model like that published in Cheng et al. (2010) and the variables related to breathing pattern (i.e., inspiratory time, respiratory frequency, and tidal volume) are not evaluated. Our group has previously analyzed and developed several models and tools in the framework of this research (Mañanas et al., 2003; Hernandez et al., 2008; Serna et al., 2010). However, although the steady-state response of such models has been thoroughly evaluated (Mañanas et al., 2003, 2004), the transient behavior had not been studied enough. For these reasons, there is a need to provide computational models that allow, with a physiological meaning, simulating a comprehensive dynamic response of the respiratory system under ventilatory stimuli like exercise.

The aim of this study is two-fold. Firstly, to propose an improved model of the respiratory system that allows simulating its dynamic response to ventilatory stimuli like exercise. Secondly, to compare its transient and static responses with those obtained from two known respiratory models (Fincham and Tehrani, 1983; Cheng et al., 2010; Cheng and Khoo, 2012) and from which the proposed model is based on. The comparison was performed by simulation and by using experimental data from healthy subjects who carried out the cardiopulmonary exercise testing (CPET). The former allowed to analyze differences among model responses and, the latter, to assess the prediction capability of each one. Due to complex structures and mechanism that may be involved during exercise, this study was focused on the dynamic response analysis of respiratory system under moderate exercise (below the lactate threshold). All models were implemented in SIMULINK/MATLAB®.

## Materials and methods

### Experimental data

A database of ten healthy male volunteers (aged 54.0 ± 13.5 years, weight 75.6 ± 10.3 kg, and height 169.9 ± 7.0 cm), non-smoking subjects, normotensive, normal lung function, and with no history of lower limb or cardiopulmonary disorders under a CPET in an electromagnetically-braked cyclorgometer (CardiO2; MedGraphics Corp., St. Paul, MN), was used in this study. The experimental protocol was carried out by a trained medical staff of the Pulmonary Function Laboratory of the Hospital Clínico de Barcelona for evaluation of exercise tolerance. This study, which was performed following the Helsinki declaration regarding the investigation with human subjects, had been previously approved by the Committee on Investigations Involving Human Subjects at the Hospital Clinic, University of Barcelona, Barcelona, Spain. Informed consent was obtained from all individual participants included in the study.

After a warming-up session consisting of five min of stretching and three min of unloaded pedaling on the cycloergometer, the exercise workload was increased by 5 or 10 W/min every minute until the subject stopped due to symptoms (i.e., dyspnea and/or leg fatigue) or no longer maintained the constant pedal rate required. The following signals were registered by the cycloergometer every 15 s: exercise workload (W), minute ventilation (${\dot{V}}_{E}$), tidal volume (*V*_*T*_), inspiration time (*T*_*I*_), expiration time (*T*_*E*_), *O*_2_ consumptions (${\dot{V}}_{O_{2}}$), *CO*_2_ productions $\left({\dot{V}}_{CO_{2}}\right)$, expired fraction of *O*_2_ (*P*_*etO*_2__) and *CO*_2_ (*P*_*etCO*_2__), and heart rate (*HR*). Taking into account that no subject had gas exchange impartments, arterial blood pressures of CO_2_ and O_2_ were adjusted considering average normal values at rest, 39.156 mmHg and 104.37 mmHg respectively (Batzel et al., 2007), as follows:

(1) $$\begin{array}{l} {\text{P}}_{{\text{aCO}}_{2}}=\;{\text{P}}_{{\text{etCO}}_{2}}+(1.78\pm 3.27) \end{array}$$

(2) $$\begin{array}{l} {\text{P}}_{{\text{aO}}_{2}}=\;{\text{P}}_{{\text{etO}}_{2}}-(1.72\pm 3.96) \end{array}$$

**Figure 1:**
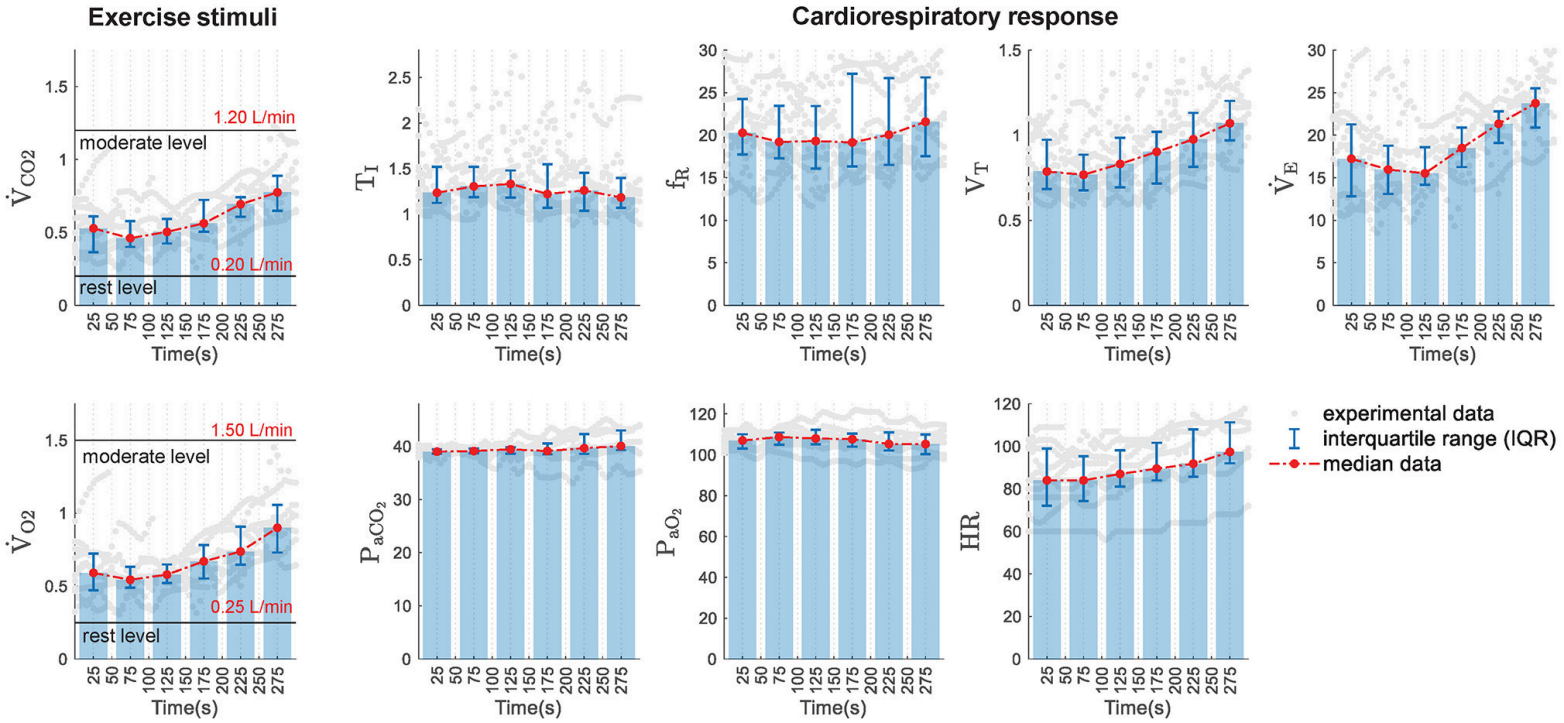
Experimental data distribution in function of time. **(Left)** Experimental exercise stimuli used to simulate the models under study: ventilated values of *CO*_2_ (${\dot{V}}_{CO_{2}}$) and *O*_2_ (${\dot{V}}_{O_{2}}$). Rest and moderate exercise levels are also marked in both variables. **(Right)** Experimental data of the cardiorespiratory variables analyzed: inspiratory time (*T*_*I*_), respiratory frequency (*f*_*R*_), tidal volume (*V*_*T*_), minute ventilation (${\dot{V}}_{E}$), arterial partial pressure of CO_2_ (*P*_*aCO*_2__) and *O*_2_ (*P*_*aO*_2__), and heart rate (*HR*). Gray dots show original experimental data, bar plots and red lines display the median values obtained in 50-s consecutive time intervals and error bars show the interquartile ranges calculated in each interval.

Figure 1 describes registered data in median and interquartile distance of the database analyzed. It can see that, excepting *T*_*I*_, *P*_*aCO*_2__ and *P*_*aO*_2__ whose values remained almost constant, all variables increased with the exercise workload. This latter was related to increments of both ${\dot{V}}_{CO_{2}}$ and ${\dot{V}}_{O_{2}}$ that, in turn, had a linear relationship with ${\dot{V}}_{E}$.

### Respiratory system models

#### RS1 model

RS1 is the respiratory model described in Fincham and Tehrani (1983) which has been extensively studied by many researchers for teaching and research purposes (Mañanas et al., 2003; Tehrani et al., 2004; Batzel et al., 2007; Hernandez et al., 2008). Additionally, it has been used to predict the effects of ventilatory settings on blood gases of mechanically ventilated patients and to adjust the ventilation parameters to optimize such a treatment both adult and infant (Tehrani and Abbasi, 2012). A schematic diagram of this model is provided in the Supplementary Material Section (see Figure S1).

RS1 includes a self-adaptive and discrete controller (Priban and Fincham, 1965), which self-adjusts the ventilation ${\dot{V}}_{E}$ and the breathing pattern, every respiratory cycle, from signals captured by different afferent pathways, as it happens physiologically. It incorporates complex peripheral processes like the gas exchange between lungs and body tissues and brain, transport delays due to blood circulation and blood gas dissociation.

To adjust ${\dot{V}}_{E}$, RS1 controller continuously receives information sent by the central (*P*_*bCO*_2__) and peripheral chemoreceptors (delayed by transport through the circulatory system, *P′_aCO_2__* and *P′_aO_2__*), computes the mean value of such pressures in each respiratory cycle and sends these signals to the ventilatory controller which calculates the alveolar ventilation, cycle to cycle, through the following expression:

(3) $$\begin{array}{l} \frac{{\dot{V}}_{A}}{{\dot{V}}_{A_{basal}}}=\;0.2332\bar{P_{bCO_{2}}}+0.2025\bar{P_{aCO_{2}}}+G_{3}+MRV+\beta \end{array}$$

where,

(4) $$\begin{array}{l} G_{3}=\{\begin{array}{c} 4.72\times 10^{-9}{\left(104-\bar{{\text{P}}_{{\text{aO}}_{2}}}\right)}^{4.9}\text{ for }\bar{{\text{P}}_{{\text{aO}}_{2}}}\leq 104\text{ Torr}\\ 0\text{ for }\bar{{\text{P}}_{{\text{aO}}_{2}}}>104\text{ Torr} \end{array}\; \end{array}$$

In this case, ${\dot{V}}_{E}$ is defined by the sum of ${\dot{V}}_{A}$ and dead space ventilation (${\dot{V}}_{D}$), where *V*_*D*_ is, in turn, a function of ${\dot{V}}_{A}$:

(5) $$\begin{array}{l} V_{D}\;=\;0.1698{\dot{V}}_{A}+0.1587 \end{array}$$

The first term of Equation (3) represents the response generated by the central chemoreceptors, where *P*_*bCO*_2__ is defined as a function depending on the brain venous and cerebrospinal fluid partial pressures of *CO*_2_ (P_vbCO_2__ and P_CSFCO_2__, respectively) and, therefore, on *P*_*aCO*_2__ (see Figure S1). The second and third terms define the response of peripheral chemoreceptors to the arterial partial pressure of *CO*_2_ and *O*_2_, respectively; the fourth term, the neural control related to metabolism or exercise and, the last one β, is a controller constant equals 17.4. ${\dot{V}}_{A_{basal}}$ is the basal alveolar ventilation for a healthy adult, and it is ~0.0673 L/s (Fincham and Tehrani, 1983).

MRV, the neural impulse derived from metabolism, represents in RS1 the neurogenic mechanism of the respiratory control system during exercise, and it is determined by the following expression:

(6) $$\begin{array}{l} \begin{array}{cc} \tau_{4}\frac{\partial }{\partial t}\frac{d\text{MRV}}{dt}=(\text{MRR}-1)-\text{MRV} & \text{for MRR}>1\;\\ \text{MRV}=0\; & \text{for MRR}\leq 1 \end{array} \end{array}$$

where MRR is the metabolic ratio defined by the current and basal of metabolic rates of the brain (*MR*_*B*_) and tissues (*MR*_*T*_), as follows:

(7) $$\begin{array}{l} \text{MRR =}\frac{{\text{MR}}_{\text{actual}}}{{\text{MR}}_{\text{basal}}}\;=\frac{{\text{MR}}_{\text{T}}(\text{actual}){+\text{MR}}_{\text{B}}(\text{actual})}{{\text{MR}}_{\text{T}}(\text{basal}){+\text{MR}}_{\text{B}}(\text{basal})} \end{array}$$

and an exercise level increment can be simulated by a change in the input RTT (the magnitude of the step defining the final value of *MR*_*T*_) which is given by:

(8) $$\begin{array}{l} \tau_{3}\frac{d{\text{MR}}_{\text{T}}}{dt}\;=\;RTT\;-\;MR_{T} \end{array}$$

In this way, Equations (6–8) allow determining the metabolic dynamics of body and brain tissues as well as the subject's neural response during exercise. These equations represent first order dynamic systems whose response rates are determined by the time constants τ_4_ = 50 and τ_3_ = 30, respectively (Fincham and Tehrani, 1983).

There are essential conditions on which Equation (3) is invalid. Under conditions of acute hypoxia associated with low levels of the arterial partial pressure of *CO*_2_, this equation can produce a negative value of ${\dot{V}}_{A}$, clearly inadmissible. In this case, apnea occurs and immediately ${\dot{V}}_{A}\;$equals zero.

Once ${\dot{V}}_{E}\;$is determined, which in turn is equal to the product of *V*_*T*_ and *f*_*R*_, breathing pattern is adjusted by the regulation of *f*_*R*_ through an optimization criterion based on minimizing the work of breathing (Otis et al., 1950), as is shown in the following expression:

(9) $$\begin{array}{l} f_{R_{Otis,\;et\;al}}\;=\frac{-E_{rs}V_{D}+\sqrt{{\left(E_{rs}V_{D}\right)}^{2}+4E_{rs}R_{rs}\pi^{2}\;V_{D}{\dot{V}}_{A}}}{2\pi^{2}R_{rs}V_{D}} \end{array}$$

where, *R*_*rs*_ and *E*_*rs*_ are the resistance and the elastance of the respiratory system, respectively. For that, the RS1 controller generates a neural signal at the beginning of each cycle such that:

(10) $$\begin{array}{l} \frac{d\nu }{dt}=\pi Asin(2\pi f_{R}t) \end{array}$$

In RS1, the blood flows control, this is, the “brain blood flow controller” and “the cardiac output controller” were modeled through algebraic relationships that allow calculating brain blood flow (${\dot{Q}}_{B}$) and total cardiac output ($\dot{Q}$) depending on partial pressures of *CO*_2_ and *O*_2_ and metabolic rate ratio (*MRR*).

### RS2 model

RS2 comprises the cardiorespiratory model described in Cheng et al. (2010) and Cheng and Khoo (2012) and it is referred by its authors as “PNEUMA.” This model is the result of the integration of key published models of the respiratory and cardiovascular system. It has been designed to simulate the cardiorespiratory control dynamic during wakefulness and sleep, so that provides realistic predictions of the physiological responses under a wide variety of conditions such as the day-to-day sleep-wake cycle, Cheyne-Stokes respiration in chronic heart failure, obstructive sleep apnea (OSA) and hypoxia-induced periodic breathing. It can be used to investigate several types of interventions: isocapnic and hypercapnic and/or hypoxemic gas administration, the Valsalva and Mueller maneuvers, and the application of continuous positive airway pressure (CPAP). The most recent version incorporates a sub-model of glucose-insulin-fatty acid regulation to simulate also the metabolic control of glucose-insulin dynamics and its interaction with the autonomic control in obese individuals (Cheng and Khoo, 2012). RS2 is available at the USC Biomedical Simulation http://bmsr.usc.edu/software/pneuma/.

RS2 has been developed using a hierarchical structure in such a way that the degree of complexity associated with each level of organization is adapted appropriately to the investigation of physiological processes at each level. This feature allows that the whole model can be presented in a compact and efficient way. RS2 is mainly composed of five principal interconnected compartments: the respiratory system, the cardiovascular system, the central control system, the sleep mechanism, and the metabolic control system. The last one allows simulating the metabolic control over the glucose-insulin dynamics and its interaction with the autonomic control in obese individuals (Cheng and Khoo, 2012). A schematic diagram of the model is given in the Supplementary Material Section (see Figure S2).

In this model, the respiratory subsystem allows the simulation of both gas exchange system and ventilatory mechanics. Unlike RS1, RS2 provides a more detailed description of chemical and physical processes generated during respiration. It includes the progressive decline of inspiratory gas pressure (*P*_*ICO*_2__and *P*_*IO*_2__), due to different sectors of anatomic dead space, using a first-order dynamic approach to calculate the arterial partial pressures of *CO*_2_ and *O*_2_ in the areas closer to the alveoli and five small serial compartments (Khoo, 1990). It also considers the phenomena of convection and dissociation of respiratory gases during cardiovascular mixing by using a second-order dynamic system that relates the arterial pressures to the alveolar pressures (Spencer et al., 1979). In general, RS2 presents more elaborate expressions to describe in greater detail the different processes that comprise respiration, as well as its control and interaction with the cardiovascular system (Cheng et al., 2010). Only the variables directly related to ventilation control are considered here for brevity, as shown below.

The ventilatory controller in RS2 incorporates the contribution of the central (*D*_*c*_) and peripheral (*D*_*p*_) chemoreceptors. In this model, the central chemoreceptors only respond to variations in the brain partial pressure of *CO*_2_ (*P*_*bCO*_2__) while the peripheral chemoreceptors are influenced by the arterial partial pressures of *CO*_2_ (P_aCO_2__) and the oxygen saturation in arterial blood (*SAO*_2_) and their multiplicative interaction (Khoo, 1990). During wakefulness, the total ventilatory demand (*D*_*T*_) is defined by the sum of the central and peripheral chemoreceptors responses as follows:

(11) $$\begin{array}{l} D_{T}=\;D_{c}+D_{p} \end{array}$$

(12) $$\begin{array}{l} D_{c}=\;\{\begin{array}{ll} {\text{G}}_{\text{c}}\;({\text{P}}_{{\text{bCO}}_{2}}-{\text{I}}_{\text{c}}\;) & \text{for }{\text{D}}_{\text{c}}\geq 0\\ \;0\; & \text{for }{\text{D}}_{\text{c}}<0\; \end{array}\; \end{array}$$

(13) $$\begin{array}{l} {\text{D}}_{p}=\{\begin{array}{c} {\text{G}}_{\text{p}}\left({\text{P}}_{{\text{aCO}}_{2}}-{\text{I}}_{{\text{pCO}}_{2}}\right)\left({\text{I}}_{{\text{pO}}_{2}}-SA{\text{O}}_{2}\right)\text{for }{\text{D}}_{\text{p}}\;\geq \;0\\ 0\text{ for }{\text{D}}_{\text{p}}<\;0 \end{array} \end{array}$$

where, *I*_*c*_, *I*_*pCO*_2__, and *I*_*pO*_2__ represent the central and peripheral chemoreceptors activation threshold and, they are equal to 45, 38, and 102.4, respectively. In this case, *P*_*bCO*_2__ is controlled by the metabolic rate (*MR*_*bCO*2_) and the brain blood flow (*Q*_*B*_) and it is defined as a function of *P*_*aCO*_2__ (Read and Leigh, 1967).

To adjust the breathing pattern, RS2 computes the respiratory frequency in function of ventilatory demand by using the following expressions (Duffin et al., 2000):

(14) $$\begin{array}{l} \begin{array}{lll} \text{if }{\text{D}}_{\text{T}}<{\text{T}}_{D}\; & \text{then} & \;f_{R}={\text{F}}_{b}\;\\ \text{if }{\text{T}}_{\text{D}}\leq {\text{D}}_{\text{T}}\leq {\text{T}}_{\text{p}}\; & \text{then} & \;f_{R}\;=\;{\text{S}}_{1\text{F}}({\text{D}}_{\text{T}}-{\text{T}}_{\text{D}})+{\text{F}}_{\text{b}}\;\\ \text{if }{\text{D}}_{\text{T}}>{\text{T}}_{\text{p}}\; & \text{then} & \text{ F }=\;{\text{S}}_{1\text{F}}({\text{T}}_{\text{p}}-{\text{T}}_{\text{D}})+{\text{S}}_{2\text{F}}({\text{D}}_{\text{T}}-{\text{T}}_{\text{p}})+{\text{F}}_{\text{b}} \end{array} \end{array}$$

where, *F*_*b*_ is the basal frequency and *T*_*D*_ and *T*_*p*_ are thresholds of *D*_*T*_ that determine the behavior of respiratory rate. For the last two options of Equation (9) the respiratory rate varies linearly respect to *D*_*T*_ with a slope established by the scaling factors *S*_1*F*_ and *S*_2*F*_, which indirectly determine the adopted ventilatory pattern by the subject (frequency and depth) depending on the level of ventilation.

Once *f*_*R*_ is determined, the neural control, derived from the respiratory centers, establishes the muscular activity integrating the total ventilatory demand and modulating it, in turn, by an auto-rhythmic and square signal. Such a signal determines each breathing cycle (*T*_*TOT*_) by using a relation 1.5:4 to define *T*_*I*_ and *T*_*E*_, as follows:

(15) $$\begin{array}{l} N\left(t\right)=\{\begin{array}{ll} \int_{0}^{{\text{T}}_{\text{I}}}{\text{D}}_{\text{T}}\text{dt} & \text{ for }0\;<\text{t}\leq 0\\ \;0 & \text{ for }{\text{T}}_{\text{I}}<\text{t}\leq {\text{T}}_{\text{TOT}} \end{array} \end{array}$$

In the case of assisted mechanical ventilation, internal neural activity is decreased. Depending on the type of ventilatory assistance, the respiratory period would be determined by the ventilator, the subject or the interaction between both.

The cardiovascular subsystem allows simulating the heart nature pulse and blood flow through the pulmonary and systemic circulations. Unlike RS1, this subsystem includes several processes like atria-ventricular mechanics, circulatory hemodynamics, SA node, change of total peripheral resistance and baroreflex (see Figure S2). Through these mechanisms, the system calculates the arterial blood pressure, ABP, heart period, HP, cardiac output, CO, and blood flow to lung for gas exchange depending on inputs from the autonomic control system, the respiratory system, and the sleep control system (Cheng et al., 2010).

Additionally, considering Equations (7, 8) of RS1, metabolic dynamics of *CO*_2_ and *O*_2_ were incorporated in RS2 to simulate exercise stimuli.

### RS3 model

A third model called RS3 has been proposed in this study to get a completed and detailed model with a more appropriate dynamic response to exercise stimuli. This model is based on RS2 to take advantages of its associated subsystems, but mainly two key features from RS1 are replaced: (a) the estimation of ventilatory demand as a function of *P*_*bCO*_2__, *P*_*aCO*_2__, *P*_*aO*_2__, and MRV and (b) the adjustment of breathing pattern by using the optimization criteria set by Otis et al. (1950).

**Figure 2:**
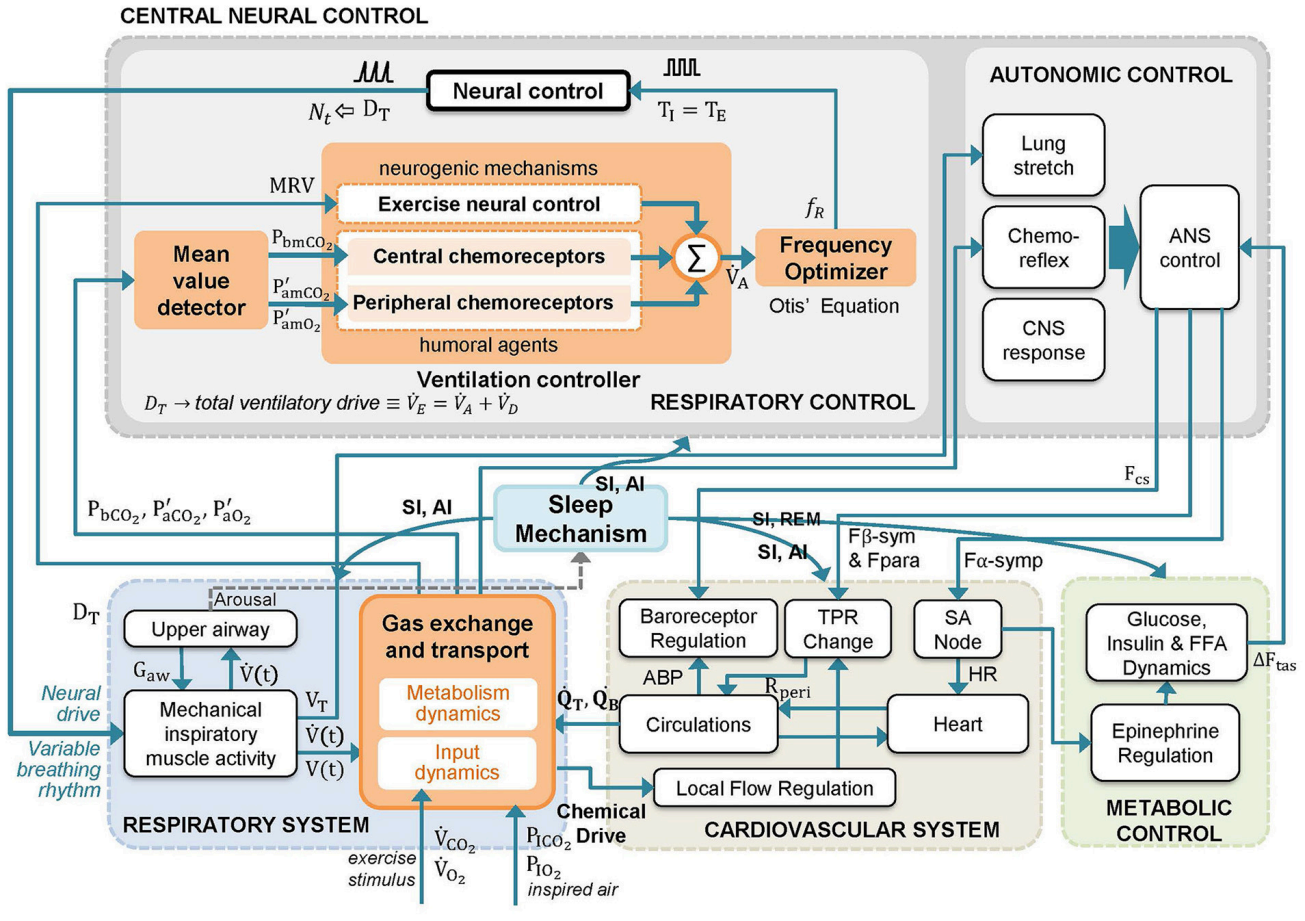
Schematic diagram of the proposed respiratory system model RS3. A model based on RS2 (Cheng et al., 2010; Cheng and Khoo, 2012) that integrates key features of RS1 (Fincham and Tehrani, 1983). The changes introduced in RS2 have been highlighted in orange. In the model, mean values of *P′_aCO_2__*, *P′_aO_2__* and *P*_*bCO*_2__ (*P*_*m*_) and a metabolically related neural drive component to the ventilation (MRV) are used by the central neural control system to compute the alveolar ventilation (${\dot{V}}_{A}$) and the total ventilatory drive ($D_{T}\equiv {\dot{V}}_{E}={\dot{V}}_{A}+{\dot{V}}_{D}$). Then, ${\dot{V}}_{A}$ is used to adjust the respiratory frequency (*f*_*R*_) each breathing cycle following the optimization principle set in Otis et al. (1950). A detailed description of model variables is provided in the Supplementary Material Section (Table S1).

Schematic diagram of RS3 is shown in Figure 2. The main changes, highlighted in orange color, are associated with the replacement of “ventilatory drive” and “respiratory rhythm” blocks of the original model RS2 by a clock pulse generator, similar to RS1 (see Figures S1, S2). The latter allows determining the onset and the end of each breath as follows: (a) the “mean value detector” block determines average values of *P*_*bCO*_2__, *P*_*aCO*_2__, and *P*_*aO*_2__; (b) the “ventilation controller” block uses previous mean values and MRV to calculate ${\dot{V}}_{A}$ by using Equation (3) and, finally, (c) the “frequency optimizer” block computes *f*_*R*_ using the Otis' Equation (Equation 9).

Moreover, three significant changes were added to the gas exchange subsystem, which is also highlighted in orange color in Figure 2, according to:

- Production of *CO*_2_ and consumption of *O*_2_ into brain tissues were included into the mass balance equations for the rate of change of the lung *CO*_2_ and *O*_2_ volumes, because they constitute ~20% of the metabolic rate at the basal level.

During inspiration,

(16) $$\begin{array}{l} {\dot{P}}_{ACO_{2}}=\;\frac{\begin{array}{r} {\dot{V}}_{T}\left(P_{d(5)CO_{2}}-P_{ACO_{2}}\right)+863\left({\dot{Q}}_{T}\left(C_{\nu CO_{2}}-C_{aCO_{2}}\right)+{\dot{Q}}_{B}\left(C_{BCO_{2}}-C_{aCO_{2}}\right)\right) \end{array}}{\left(V_{LCO_{2}}+V_{T}\right)} \end{array}$$

(17) $$\begin{array}{l} {\dot{P}}_{AO_{2}}\;=\;\frac{\begin{array}{r} {\dot{V}}_{T}\left(P_{d\left(5\right)O_{2}}-P_{AO_{2}}\right)+\;863\left({\dot{Q}}_{T}\left(C_{\nu O_{2}}-C_{aO_{2}}\right)+{\dot{Q}}_{B}\left(C_{BO_{2}}-C_{aO_{2}}\;\right)\right) \end{array}}{\left(V_{LO_{2}}+V_{T}\right)} \end{array}$$

During expiration,

(18) $$\begin{array}{l} {\dot{P}}_{ACO_{2}}=\frac{-\;863\left({\dot{Q}}_{T}\left(C_{\nu CO_{2}}-C_{aCO_{2}}\right)+{\dot{Q}}_{B}\left(C_{BCO_{2}}-C_{aCO_{2}}\;\right)\right)}{\left(V_{LCO_{2}}+V_{T}\right)} \end{array}$$

(19) $$\begin{array}{l} {\dot{P}}_{AO_{2}}=\frac{-\;863\left({\dot{Q}}_{T}\left(C_{vO_{2}}-C_{aO_{2}}\right)+{\dot{Q}}_{B}\left(C_{BO_{2}}-C_{aO_{2}}\;\right)\right)}{\left(V_{LO_{2}}+V_{T}\right)} \end{array}$$

where *V*_*LCO*_2__ and *V*_*LO*_2__ denote the volume storage of *CO*_2_ and *O*_2_ in the lungs. Likewise, *Ṗ*_*ACO*_2__ and *Ṗ*_*AO*_2__ represent the alveolar partial pressures of *CO*_2_ and *O*_2_; *C*_*aCO*_2__, *C*_*aO*_2__, *C*_*vCO*_2__, and *C*_*vO*_2__ symbolize the arterial and venous concentrations in body tissues of these gases, respectively; *C*_*BCO*_2__, *C*_*BO*_2__ denote the brain concentrations and *Q*_*T*_ and *Q*_*B*_ the blood rate in body and brain tissues.

- The exchange in the brain considered as follows:

(20) $$\begin{array}{l} \left({\text{V}}_{{\text{BCO}}_{2}}+{\text{V}}_{\text{T}}\right){\dot{\text{C}}}_{{\text{BCO}}_{2}}={\dot{\text{Q}}}_{\text{B}}({\text{C}}_{{\text{aCO}}_{2}}-{\text{C}}_{{\text{BCO}}_{2}})\;+\;{\text{MR}}_{{\text{BCO}}_{2}} \end{array}$$

(21) $$\begin{array}{l} \left(V_{BO_{2}}+V_{T}\right){\dot{C}}_{BO_{2}}=\;{\dot{Q}}_{B}\left(C_{aO_{2}}-C_{BO_{2}}\right)+MR_{BO_{2}} \end{array}$$

where *V*_*BCO*_2__ and *V*_*BO*_2__ denoted volume storage of *CO*_2_ and *O*_2_ in the brain.

- Calculation of *V*_*D*_ as a function of ${\dot{V}}_{A}$ (see Equation 5).

These changes provide to RS3 a more detailed gas exchange plant, and although they do not improve its transient response, they provide additional information that can be useful in future studies connected to, for example, analysis of brain-tissues relationship implicated in the pulmonary gas exchange.

Furthermore, to guarantee that ventilation generated by the mechanical plant matched ventilatory demand, the neural signal *N*(*t*) in RS3 was adjusted through the following expression:

(22) $$\begin{array}{l} N\left(t\right)=\{\begin{array}{ll} K\int_{0}^{{\text{T}}_{I}}{\text{D}}_{\text{T}}\text{dt} & \text{for }0\;<\text{t}\leq 0\\ \;0 & \text{for }{\text{T}}_{\text{I}}<\text{t}\leq {\text{T}}_{\text{TOT}} \end{array} \end{array}$$

where,

(23) $$\begin{array}{l} K\;=\left(\frac{\dot{V}\left(T_{I}\right)+b^{V\left(T_{I}\right)}}{b^{V\left(T_{I}\right)}-0.25\dot{V}\left(T_{I}\right)}\right)\left(\frac{RC}{D_{T}T_{I}}\right)\times \left(E_{rs}V\left(T_{I}\right)+R_{rs}\dot{V}\text{​​}\left(T_{I}\right)\right)e^{V\left(T_{I}\right)/0.28VC} \end{array}$$

RC denotes the muscle constant time (0.060s) and VC the vital capacity (5 L).

Like in RS1 and RS2, metabolism dynamic of *CO*_2_ and *O*_2_ was incorporated in the gas exchange plant with the aim of simulating exercise stimuli, see Equations (7, 8).

### Simulation

Responses and features of the three models were evaluated considering different levels of exercise. In this stimulus, the consumption of *O*_2_ and the production of *CO*_2_ rise significantly increasing the ventilated values of *CO*_2_ and *O*_2_. For this reason, a step input of ${\dot{V}}_{CO_{2}}$ and ${\dot{V}}_{O_{2}}$ from rest (0.20 and 0.25 L/min), to moderate exercise (1.20 and 1.50 L/min), under conditions of normoxia, was considered to analyze their transient responses. Then, similarly, 11 equidistant step inputs among such intervals were taken into account to evaluate their stationary responses. These values were selected considering those published in Mañanas et al. (2002) and Guyton (2015) for moderated exercise and experimental data obtained in the CPET test (see Figure 1). Additionally, a sensitivity analysis was carried out with RS3 to assess the individual roll of the neurogenic and neuro-humoral mechanisms implemented to simulate exercise.

### Prediction error

To assess the prediction capability of each model, experimental values of ${\dot{V}}_{CO_{2}}$ and ${\dot{V}}_{O_{2}}$ were used to simulate exercise stimulus. Then, the output cardiorespiratory variables predicted by the models were analyzed concerning the ones obtained experimentally. Comparison of each model response regarding experimental data was evaluated quantitatively by the prediction error (PE) calculated from the following variables:

- ${\dot{V}}_{E}$, *T*_*I*_, *f*_*R*_, and *V*_*T*_, which provide information about ventilatory strategy or breathing pattern adopted for each model (controller) to adjust ventilation,
- HR, which provides information about cardiac activity, and
- *P*_*aCO*_2__ and *P*_*aO*_2__, which allow assessing the regulation of *CO*_2_ and *O*_2_ respectively.

PE was calculated by measuring percent differences between simulated, SIM, and experimental, EXP, variable as follows:

(24) $$\begin{array}{l} PE\left(\%\right)=100\times \frac{1}{k}\sum_{i\;=\;1}^{k}\left|\frac{\nu ar_{EXP\left(i\right)}-\nu ar_{SIM\left(i\right)}}{\nu ar_{EXP\left(i\right)}}\right| \end{array}$$

where k denotes the number of samples. Overall prediction error was obtained averaging the PE for all variables.

Regarding HR, due to RS1 does not provide direct information about it, this variable was obtained indirectly from the cardiac output (Q), considering a constant stroke volume (SV = 70 mL) and using the expression HR = Q/SV (Fincham and Tehrani, 1983; Batzel et al., 2007). Although this is a simple approximation, it allows comparing the cardiac response of RS1 with the other models.

### Statistical analysis

Non-parametric tests, Friedman and Wilcoxon-Mann-Whitney (WMW), were used to identify statistical differences between prediction capability with a significance level of ρ = 0.05. The former was used in order to find differences between the model errors, and the latter to identify the model with the best fitting to experimental data. Each simulation was run once due to models are determinists (i.e., their responses do not change if the initial conditions and stimulus step size remain unchanged).

### Availability

The models RS1, RS2, and RS3 can be interactively tested through a Matlab app, which is available at https://bioart.upc.edu/en/virtual-laboratories/modules upon query. Additionally, median values and interquartile distances of experimental data analyzed in this study as well as the stimulus levels used during simulation models are also provided.

## Results

### Simulations

#### Transient response

**Figure 3:**
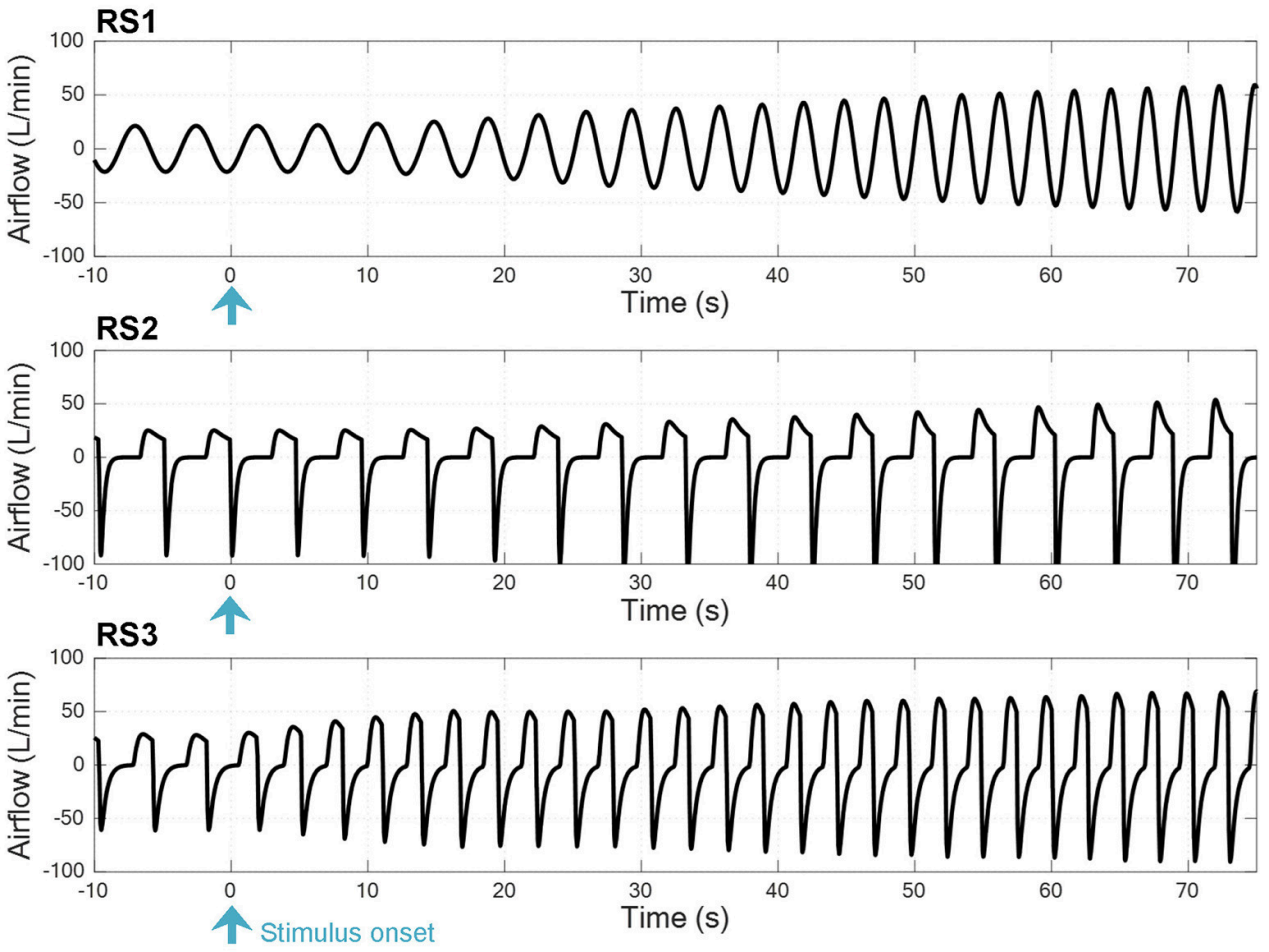
Time series breath-to-breath of airflow signal at the onset of exercise when a step input was used to simulate the respiratory system models RS1, RS2, and RS3 (see text).

Figure 3 shows a time series breath-to-breath of airflow signal for each model at the onset of exercise when a step input of ${\dot{V}}_{CO_{2}}=$1.25 L/min and ${\dot{V}}_{O_{2}}=$1.50 L/min was applied to simulate each model (0.2 and 0.25 L/min values were considered as rest levels, respectively). In all models, it can be seen an increase in ventilation, which is higher and faster in RS3.

**Figure 4:**
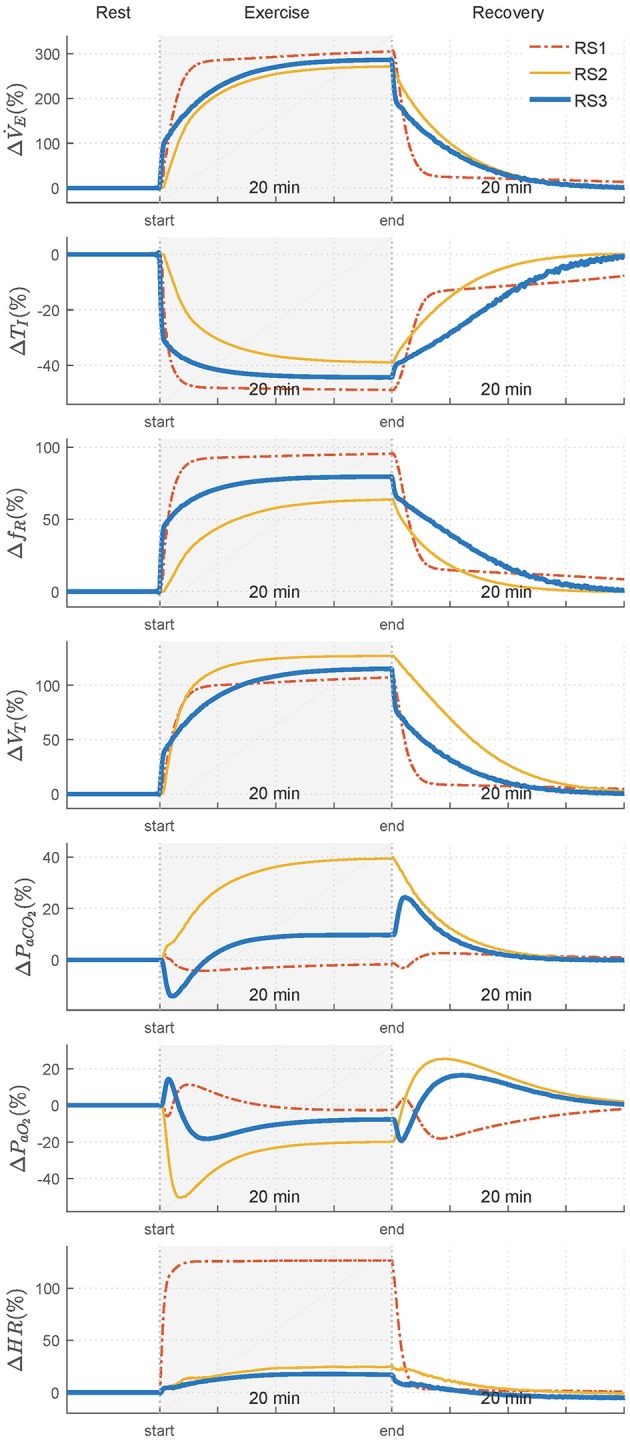
Transient responses of analyzed variables—ventilation (${\dot{V}}_{E}$), inspiratory time (*T*_*I*_), respiratory frequency (*f*_*R*_), tidal volume (*V*_*T*_), arterial *CO*_2_ pressure (*P*_*aCO*_2__), arterial *O*_2_ pressure (*P*_*aO*_2__), and heart rate (*HR*)—when a step exercise input (${\dot{V}}_{CO_{2}}=$ 1.20 L/min and ${\dot{V}}_{O_{2}}\;=$1.50 L/min) was used to simulate RS1, RS2, and RS3. Variations of each variable are showing regarding their respective values in rest.

Figure 4 shows the transient response obtained by the models. An additional interval, after of the stimulus, was also considered to simulate the recovery phase. Values of each variable are shown regarding their basal or rest values. Table 1 shows the constant times obtained in each of them.

**Table 1:** Rise time (τ) of the dynamic response of RS1, RS2, and RS3 obtained when a step exercise input of ${\dot{V}}_{CO2}=$1.20 L/min and ${\dot{V}}_{O2}=$1.50 L/min was used to simulate them.

**Variable**	τ (**mm:ss**)		
	**RS1**	**RS2**	**RS3**
*T*_*I*_	00:38	03:00	00:45
*f*_*R*_	00:52	04:15	01:15
*V*_*T*_	01:24	01:55	02:45
${\dot{V}}_{E}$	01:22	03:15	02:40
*P*_*aCO*_2__	00:57	04:20	03:30
*P*_*aO*_2__	00:44	00:55	01:20
*HR*	00:21	04:55	03:25

Regarding variables related to breathing pattern, *T*_*I*_, *f*_*R*_, and *V*_*T*_, for RS1 and RS2, an exponential behavior was observed both exercise and recovery, being RS1 faster than RS2. For RS3, such responses were characterized by two main temporal phases during exercise: an initial phase determined by an instantaneous increase of almost 100% of ${\dot{V}}_{E}$ and a second phase defined by a gradual increase of ${\dot{V}}_{E}$ to its steady state-value. During recovery, ${\dot{V}}_{E}$ and *V*_*T*_ presented a similar behavior and opposite to that obtained during exercise while *f*_*R*_ and, therefore, *T*_*I*_ were given by the dynamics defined in Equation (9) (Otis et al., 1950).

Regarding the variables related to gas exchange and for RS1, *P*_*aCO*_2__ presented initially a slight overshoot, which was followed by a decrease and subsequent exponential evolution toward a value close to its basal level. By contrast, *P*_*aO*_2__ exhibited an initial slight drop, which was followed by a positive overshoot of about 10% and an exponential evolution that, as *P*_*aCO*_2__, converged to a value close to that obtained during rest. This behavior was also reported by the same authors in Fincham and Tehrani (1983). For RS2, *P*_*aCO*_2__ increased exponentially until overcomes its basal value by 40% (≈56 mmHg), while *P*_*aCO*_2__ rapidly declined to 50% (≈50 mmHg), reaching values close to hypoxia (*P*_*aCO*_2__ < 70 mmHg) (Roussos and Koutsoukou, 2003), for then evolves exponentially at a steady-state value of 80 mmHg.

For RS3, *P*_*aCO*_2__ was characterized by an initial decrease, of about 15% from its basal value, followed by an exponential growth toward a steady-state value 10% higher than its basal value. *P*_*aO*_2__ was defined by, first, an overshoot that, after ~1 min, evolved almost exponentially toward its stationary value with a negative overshoot that was not as critical as that found in RS2. During recovery, both *P*_*aCO*_2__ and *P*_*aO*_2__ presented similar and opposite behaviors in all models. In addition, evolution of *P*_*aCO*_2__ was slower than *P*_*aO*_2__, especially for RS2 and RS3 (see Table 1), possibly due to the larger storage capacity available for *CO*_2_ (*V*_*TCO*_2__ = 15 L) compared to *O*_2_(*V*_*TO*_2__ = 6 L). A similar behavior has been reported in Mateika and Duffin (1995).

Finally, for all models, HR exponentially evolved with different velocities toward its final value. Particularly for RS1, the response time of HR was lower than that obtained by the other models, and its increase from baseline was quite higher (≈170 bpm) than that expected for moderate exercise (85–110 bpm), see Figure 1.

### Steady state response

**Figure 5:**
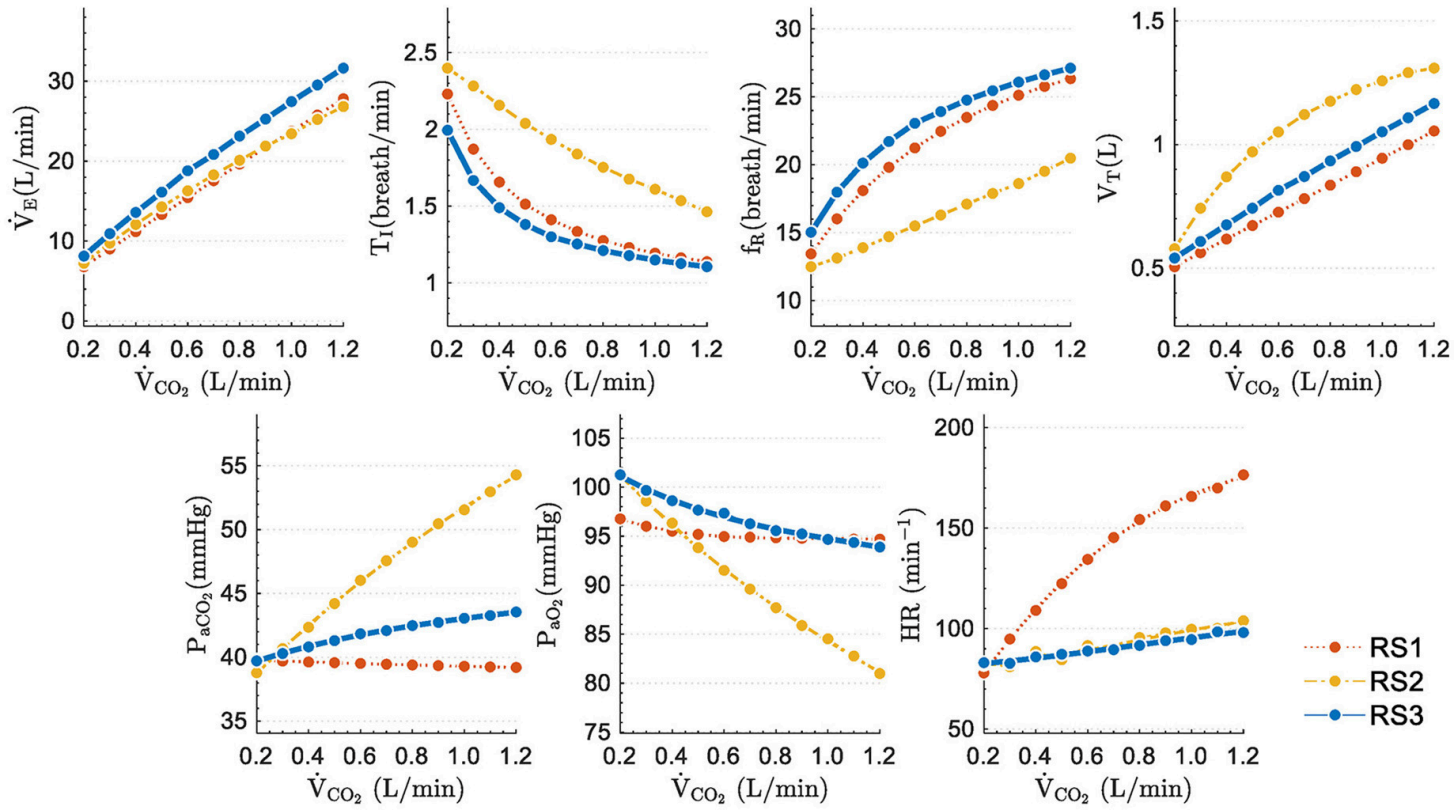
Steady state values obtained when step inputs of exercise from 0.25 to 2.50 L/min of ${\dot{V}}_{CO_{2}}$ were used to simulate the respiratory system models under study.

Figure 5 shows the final values of analyzed variables in function of different levels of exercise. Eleven step inputs from 0.2 to 1.2 L/min for ${\dot{V}}_{CO_{2}}$, with *P*_*IO*_2__ fixed to its sea level value (150 mmHg), were applied to simulate the models and analyze their steady-state responses under moderated exercise.

Excepting *P*_*aCO*_2__, variables of all models increased followed similar trends: *T*_*I*_ and *P*_*aO*_2__ decreased while *f*_*R*_, *V*_*T*_, and ${\dot{V}}_{E}$increased with higher stimuli. Static performance in RS1 and RS3 for *T*_*I*_, *f*_*R*_, *V*_*T*_, and ${\dot{V}}_{E}$ was quite similar since both models include the same neural ventilatory control to calculate ${\dot{V}}_{E}$, optimize *f*_*R*_ and, therefore, set *T*_*I*_ and *V*_*T*_.

The slight differences found among the model responses were mainly due to: (a) model basal values no related to exercise, (b) differences among the gas exchange plant, and (c) the control systems implemented in each model. The steady-state values of *P*_*aCO*_2__ and *P*_*aO*_2__ further evidence these differences. RS1 and RS3 were able properly to regulate *P*_*aCO*_2__ and *P*_*aO*_2__, while RS2 converged to values very different from those expected during moderate exercise (*P*_*aCO*_2__ ≈ 40 mmHg and *P*_*aO*_2__ ≈ 104 mmHg) (Guyton, 2015). For RS1 and RS3, the proper regulation of such gases could be because ${\dot{V}}_{E}$ was adjusted to match the ventilatory demand generated by exercise.

Regarding HR, in RS2 and RS3, this variable raised slightly and linearly with the stimulus level, reaching approximately an increase of 20% at the highest stimulus. On the contrary, in the RS1 model, HR increased considerably (up to 130%), moving away from the expected range for this type of stimulus.

### Analysis of RS3 control mechanisms

**Figure 6:**
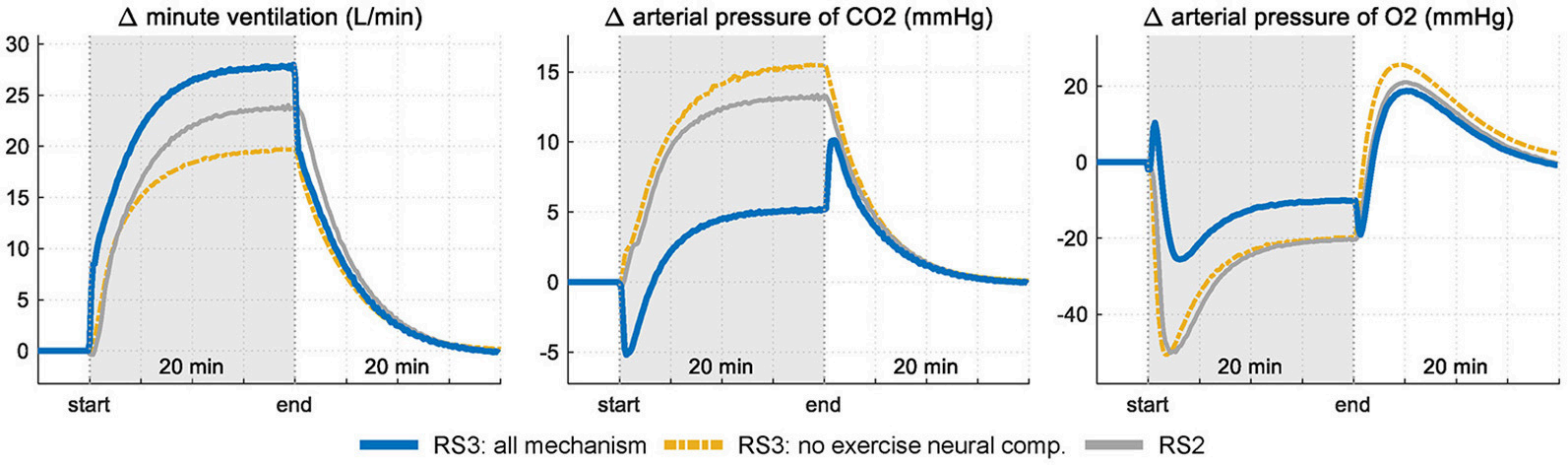
Response of RS3 to a step exercise input under two conditions: firstly, with all mechanism of respiratory control operative and, secondly, without the neurogenic mechanism MRV (see Equation 3). Results are compared with those obtained in RS2 for the same stimulus. Variations of each variable are showing regarding their respective rest values.

Figure 6 shows the results obtained in the sensitivity analysis carried out in RS3. ${\dot{V}}_{E}$, *P*_*aCO*_2__ and *P*_*aO*_2__ are shown in two different conditions: first, with all mechanisms working and second, without the neurogenic mechanism MRV (exercise neural control component). Results are compared with those obtained with RS2.

Selective elimination of neurogenic mechanism MRV leads to a slower increase of ${\dot{V}}_{E}$ during exercise with a consequent and important drop of *P*_*aO*_2__ at the onset of exercise (up to 50 mmHg) and an exponential increase in *P*_*aCO*_2__ (up to 12 mmHg from baseline). During the stimulus, the final ventilation value of RS3 differs only by 5 L/min from the value reached when all the mechanisms are considered, case in which *P*_*aCO*_2__ and *P*_*aO*_2__ vary slightly from their values at rest. This is because the absence of a neurogenic mechanism is compensated by central and peripheral chemoreceptors, which are greatly stimulated by the large decompensation of arterial pressures, especially *P*_*aCO*_2__. On the other hand, the action of the humoral mechanism (central chemoreceptors response) of RS2 generates a ventilation inferior to that obtained by RS3, a fact that contributed to a greater increase of *P*_*aCO*_2__ for the same stimulus level.

**Figure 7:**
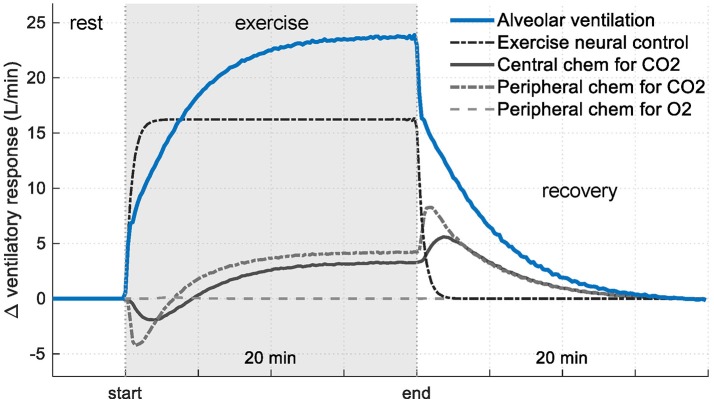
Contribution of each controller component of RS3 on ventilatory response (see Equation 3). Results are shown regarding their respective rest values.

Figure 7 shows the contribution from each controller component of RS3 regarding its basal value. In this case, it can be seen that, during exercise, the presence of the exercise neural control component (neurogenic mechanism) generates a change in ventilation of up to 16 L/min (67%) while central and peripheral chemoreceptors contribute only with 8 L/min (33%) in the total ventilation. For the latter, this contribution is mainly generated by the changes given in *P*_*aCO*_2__ due to the relationship between ${\dot{V}}_{A}$ and *P*_*aCO*_2__ (i.e., changes in ${\dot{V}}_{A}$ by *P*_*aO*_2__ only are significant when this is lower than ≈ 60 mmHg, see Equation 4).

### Experimental data

**Figure 8:**
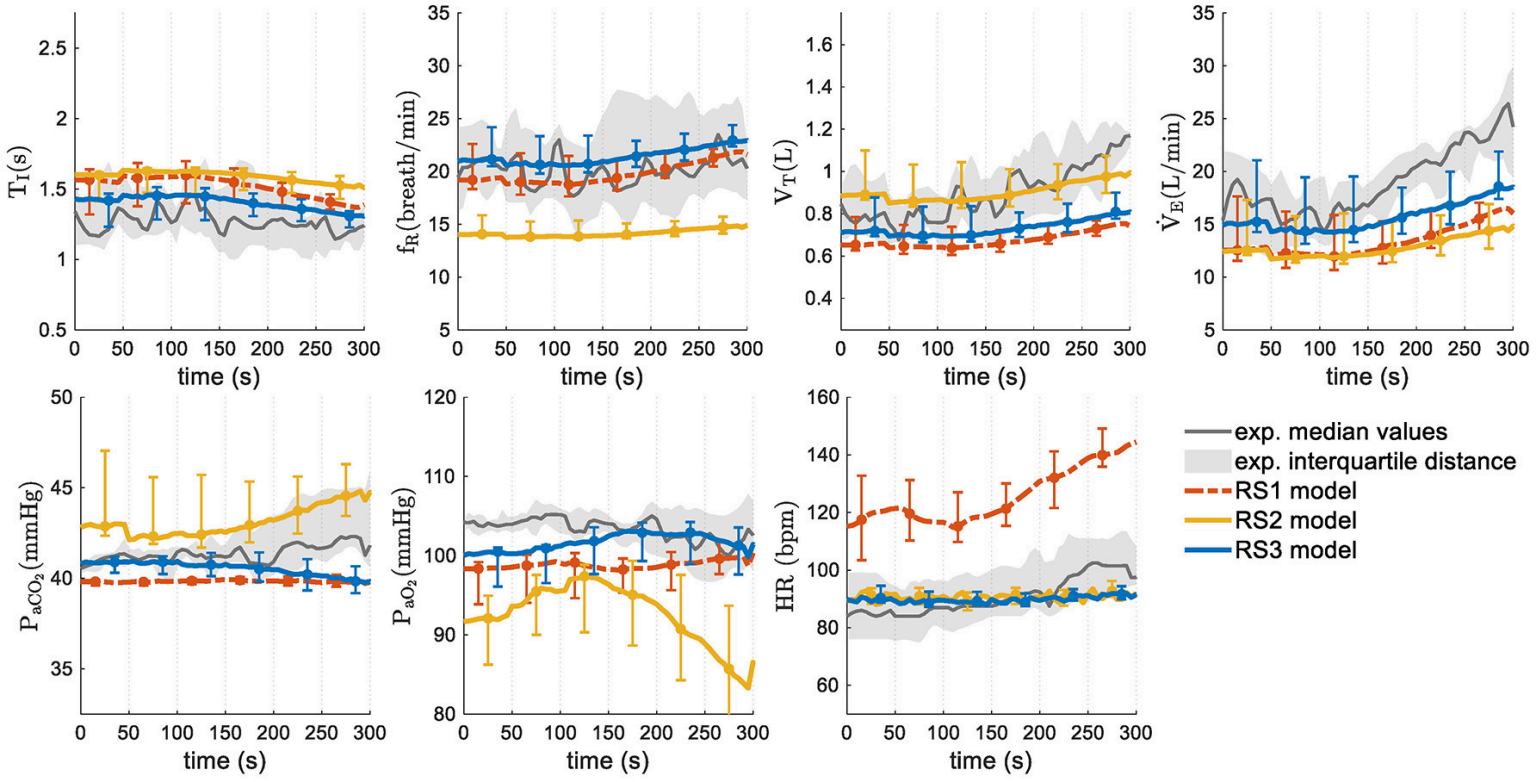
Transient responses of models analyzed in this study. Median values and interquartile distance of experimental and simulated data are presented for each variable and model. Gray shadows show the IQR of experimental data and error bars the IQR of simulated data obtained by each model.

Given the nature of the stimuli used to simulate the models from experimental data (a progressive increase, contrary to step function), only responses in “transient regime” were analyzed. Figure 8 shows simulation results in median and interquartile distance. In this case, both experimental data and model responses of ${\dot{V}}_{E}$, *f*_*R*_, and *V*_*T*_ increased with the increment of exercise stimulus whereas *T*_*I*_ decreased slightly. Regarding *P*_*aCO*_2__ y *P*_*aO*_2__, RS1 and RS3 showed an appropriate regulation. For RS2, these latter variables took values a little apart from experimental data at the highest stimulus levels. Respect to HR, RS2 and RS3 showed a good fitting whereas RS1 presented values far away of experimental data.

**Figure 9:**
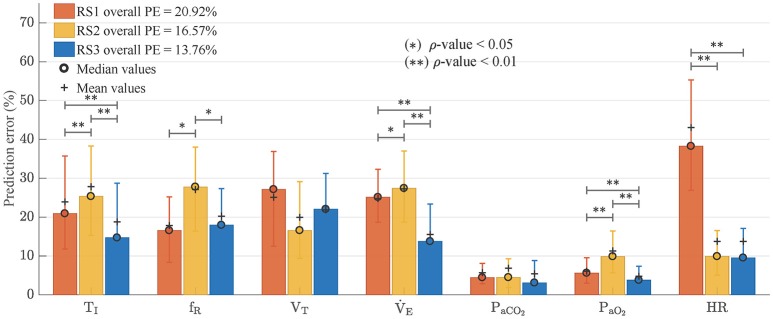
Prediction errors (PE) obtained by each one of analyzed models. Bars graph shows median values and error bars indicate interquartile distance (range of values from 25 to 75% quartile). Wilcoxon signed-rank test was used to find statistically significant differences between the obtained PE. (^*^) for ρ < 0.05 and (^**^) for ρ < 0.01.

Figure 9 shows the median values and interquartile distance of prediction error calculated from Equation (24). Friedman test showed statistically significant differences in all variables except *f*_*R*_ and *V*_*T*_, where the ρ-values were 0.149 and 0.122 respectively. In general, RS3 was the model with the best adjustment. It reached the lowest overall PE (13.76 vs. 20.92% for RS1 and 16.57% for RS2). Wilcoxon signed-rank test showed statistical differences between RS3 and the other models (ρ < 0.01) whereas RS1 and RS2 did not have any statistically significant difference (ρ = 0.432). Regarding each variable, RS3 presented the best fitting for ${\dot{V}}_{E}$, *T*_*I*_, *P*_*aO*_2__, *P*_*aCO*_2__, and HR with ρ < 0.01 for the first three variables. RS1 and RS2 showed a better adjustment for *f*_*R*_ and *V*_*T*_, respectively, but without any significant statistical difference respect to RS3 (see Figure 9).

On the other hand, HR in RS1, which was calculated from ${\dot{Q}}_{T}$ and a stroke volume of 70 mL, presented a very high prediction error. In this case, the absence of a more complex cardiovascular subsystem impeded a suitable simulation of HR response to exercise.

## Discussion

Dynamic responses of three respiratory system models under exercise stimuli have been analyzed in this study. The performances of these models were evaluated by using two settings: first, through simulation to assess the realism of the dynamics of their responses and second, using experimental data to estimate the prediction capability of each model.

### Dynamic response

#### Transient response

Transient response of each model was evaluated by using a moderate exercise step input. In this case, **RS1** presented a relatively fast response at the onset of exercise (see Table 1), with an exponential response for ${\dot{V}}_{E}$ and an appropriate regulation for *P*_*aCO*_2__ and *P*_*aO*_2__ at the end of the stimulus. **RS2** presented an exponential growth in ${\dot{V}}_{E}$ that was not enough to regulate the arterial gases. Due to this, *P*_*aO*_2__ decreased dramatically, during the transient phase reaching hypoxemic values (*P*_*aO*_2__ ≈ 50 mmHg) and *P*_*aCO*_2__ increased by 40% its basal value reaching hypercapnic values (*P*_*aCO*_2__ ≈ 56 mmHg). This fact invalidates its simulation for this kind of stimulus. Finally, **RS3** presented at the onset of exercise an sudden increase of ${\dot{V}}_{E}$ which was followed by an intermediate phase characterized by an exponential growth, and a final phase defined by its static value. The “abrupt” increase of ${\dot{V}}_{E}$ caused *P*_*aCO*_2__ to decrease slightly and that *P*_*aO*_2__ obtained an overshoot at the beginning of the stimulus. Then, in the intermediate phase, *P*_*aO*_2__ decreased while *P*_*aCO*_2__ raised as a result of the increase in consumption of *O*_2_ and production of *CO*_2_, respectively, and its dissociation with ${\dot{V}}_{E}$ (Whipp and Ward, 1991) and, at the final phase, *P*_*aCO*_2__ and *P*_*aO*_2__ took values near their basal values (see Figure 4).

Regarding variables involved in breathing pattern (*T*_*I*_, *f*_*R*_, and *V*_*T*_), it was found that, except RS3 at the onset of the stimulus, all variables evolved similarly and exponentially toward their steady-state values. Differences found among them were mainly given by the ventilatory controller implemented in each model. On the other hand, behavior of HR in RS2 and RS3 was very similar, and its values were within the range defined for moderate exercise, contrary to RS1, where HR took values far away to those expected (see Figures 1, 4).

#### Static response

The steady-state model responses were evaluated considering a sequence of step inputs from rest to moderate exercise. In all models, ${\dot{V}}_{E}$ linearly increased with the stimulus level (see Figure 5). Changes between consecutive stimulus were quite similar. Respect to *f*_*R*_, this variable also linearly augmented in RS2 according to Equation (14), while in RS1 and RS3 it was determined by Equation (9), which guarantees the minimum respiratory work of breathing (Otis et al., 1950). These adjustments affected the behavior of *T*_*I*_ and *V*_*T*_ due to their relationship with ${\dot{V}}_{E}$ and *f*_*R*_ (${\dot{V}}_{E}\;=f_{R}\times V_{T}=V_{T}/T_{I}+T_{E}$).

On the other hand, RS1 and RS3 presented an appropriate regulation for *P*_*aCO*_2__ y *P*_*aO*_2__ (the final values were closer to normal ones at rest, *P*_*aCO*_2__ ≈ 40 mmHg and *P*_*aO*_2__ ≈ 104 mmHg) (Guyton, 2015). Particularly in RS3, *P*_*aCO*_2__ and *P*_*aO*_2__ shown slight variation regarding their basal values such has been reported from several studies (Krogh and Lindhard, 1913; Pearce and Milhorn, 1977; Magosso and Ursino, 2005). RS2 did not show a proper regulation of the arterial gases due to *P*_*aCO*_2__ increased toward hypercapnic values (*P*_*aCO*_2__ ≈ 54 mmHg) while *P*_*aO*_2__ decreased toward hypoxemic values (*P*_*aO*_2__ ≈ 80 mmHg), lower than expected ones (Guyton, 2015).

### Control mechanisms

In RS3, the abrupt increase of ${\dot{V}}_{E}$ at the onset of exercise and, therefore, the initial changes in *P*_*aCO*2_ and *P*_*aO*2_, was mainly determined by the “MRV” component incorporated in its controller (see Equation 4). This component played an important role in the determination of anticipatory ventilatory response and allowed simulating, with a straightforward approach, the neurogenic mechanism of ventilation to exercise, i.e., the so-called central command (direct activation of the respiratory control centers by the locomotor stimulus), chemoreceptors in large vessels and mechanoreceptors located in exercising muscles (Dempsey and Smith, 2014), see Figures 6, 7. Although, an only “exercise” component in the respiratory controller could not be enough to simulate each underlie mechanism to this type of control, similar results to those obtained in RS3, especially with regard to the behavior of ${\dot{V}}_{E}$, *P*_*aCO*_2__, and *P*_*aO*_2__, have been reported in several sources (Krogh and Lindhard, 1913; Pearce and Milhorn, 1977; Whipp et al., 1982; Mateika and Duffin, 1995; Turner et al., 1997; Whipp and Ward, 1998; Magosso and Ursino, 2005; Wasserman et al., 2011; Parkes, 2013; Guyton, 2015).

Particularly, the changes in ventilation, blood flow, pulse rate, respiratory exchange and alveolar *CO*_2_ tension, which take place in men during the first minute of light (moderate) or heavy work, presented in Krogh and Lindhard (1913) agree with the transient responses obtained in RS3, see Figure 4. They found in all the cases examined (six subjects, three of them subject trained to sudden exertions) a sudden rise in ventilation when an exercise step was used to stimulate the subjects. This abrupt increase was greater with heavier work. Although differences were found between trained and no-trained subjects, the latter presented a higher increase in ${\dot{V}}_{E}$ that, although was not as pronounced as in trained subjects, was always present even during moderate exercise. Regarding gases regulation, as in RS3 a considerable fall in alveolar *CO*_2_ tension was detected, which was, likewise, followed by a *CO*_2_ increase. A similar behavior was also reported in Parkes (2013). They found a nearly exponential increment of ventilation during exercise, while *P*_*aO*_2__ decreased regarding its rest value at the beginning of stimulus for, then, growing up exponentially to its steady state. Moreover, the simulated data reported in Magosso and Ursino (2005), which were found according to experimental dynamic responses of human subjects to bicycle exercise published in Pearce and Milhorn (1977), were also consistent with RS3.

It is important to note that respiratory drive and therefore, ventilatory response (${\dot{V}}_{A}$) in RS3 (and RS1) is related to *P*_*bCO*_2__, *P*_*aCO*_2__, *P*_*aO*_2__ and *MRV* through Equation (3). Thus, there is a linear relationship with *P*_*bCO*_2__, *P*_*aCO*_2__, and MRV and an exponential dependence with *P*_*aO*_2__ (see Equation 4). So, unitary changes in *P*_*bCO*_2__, *P*_*aCO*_2__, and MRV would modify ${\dot{V}}_{A}$ by a quantity of 0.2332, 0.2025, and 1 of its basal value (${\dot{V}}_{A_{basal}}$). By contrast, decreases of P_aO2_ would exponentially increase ${\dot{V}}_{A}$ only if *P*_*aO*_2__ < 104.9 (see Figure 7).

On the other hand, in this study, exercise stimulus was used to generate different levels of ventilation and evaluate how the models adjust ventilation and breathing pattern to accomplish metabolic demand and regulate arterial blood gases. RS1 and RS3 used an optimization principle that allows adjusting *f*_*R*_ by minimizing work of breathing. In this sense, the minimization of WOB has been extensively considered as a control criterion to adjust the breathing pattern (Yamashiro and Grodins, 1971; Poon et al., 1992; Serna Higuita et al., 2014; Serna et al., 2016). Moreover, recent formulations seek to describe how sensory information influences the dynamics of respiratory rhythm under the hypothesis that “respiratory rhythms arise from the interplay of central rhythm generation circuits, biomechanics and feedback from peripheral signaling pathways” (Butera et al., 1999a,b; Diekman et al., 2017). Particularly, rhythmogenesis is investigated in Diekman et al. (2017) in a simple model of close-loop control, incorporating biomechanics, oxygen handling, metabolism and chemo-sensation. In such study, the Butera-Rinzel-Smith model (Butera et al., 1999a) of bursting pacemaker neurons in the preBötzinger complex is adopted as their central pattern generator. Although, peripheral processes modeled in Diekman et al. (2017) are not as comprehensive as RS1, RS2, and RS3, this approach to simulate the dynamics of rhythm respiratory could be interesting to provide RS3 a more realistic pattern generator.

Finally, highly regulated neural inputs are critical to maintaining normal cardiovascular function. Although the cardiovascular central command during exercise is typically associated with a perception of effort, there is not a clear understanding of the role of central command in the integration of sensory information that can define more completely the relevance of central command for the neural control of exercise (Williamson, 2010).

### Prediction capability

Regarding the model goodness of fit, RS3 presented the overall best adjustment to experimental data with the lowest prediction error and an improvement of 17% respect to RS2 (overall PE = 13.51%, see Figure 9). RS3 also presented the lower prediction errors for *T*_*I*_, ${\dot{V}}_{E}$, *P*_*aCO*_2__, *P*_*aCO*_2__, and HR with statistically significant differences for the first three.

While the reduction of the prediction error in RS3 (13.76%) is lower concerning RS2 (16.57%) than to RS1 (20.92%) when using experimental data, in both cases, these reductions were statistically significant (ρ < 0.01). This improvement is more evident if only the respiratory variables *T*_*I*_, *f*_*R*_, *V*_*T*_, and ${\dot{V}}_{E}$ are considered. In this case, the prediction errors are 23.0, 26.4, and 16.3%, for RS1, RS2, and RS3 respectively, and RS3 presents an improvement of 38% regarding RS2. Such difference is due to prediction errors of *P*_*aCO*_2__ and *P*_*aO*_2__in all models were relatively small by the magnitude of these variables.

## Conclusion

Three respiratory system models have been analyzed in this paper. Two of them published Fincham and Tehrani (1983), Cheng et al. (2010), and Cheng and Khoo (2012), named RS1 and RS2 respectively, and the other one is a model proposed in this study, called RS3 and based on the integration of key features of the first two.

The first analyzed model, RS1 (Fincham and Tehrani, 1983), is a complex model that adjusts ${\dot{V}}_{E}$ and the breathing pattern by minimizing the work of breathing through regulation of *f*_*R*_ (see Equation 9) (Otis et al., 1950). It integrates several peripheral processes and self-adjust the ventilation and breathing pattern at the end of each breath from signals captured by humoral and neurogenic afferent pathways. Simulation of this model, under exercise stimuli, showed a good adjustment of ${\dot{V}}_{E}$ and a proper regulation of arterial gases (*P*_*aCO*_2__ and *P*_*aO*_2__). This was the key feature that motivated us to use the neural controller of RS1 in RS3.

The second model analyzed, RS2 (Cheng et al., 2010; Cheng and Khoo, 2012) is a more comprehensive model. It integrates the interaction between the respiratory and cardiovascular systems and allows simulating the dynamic of cardiorespiratory control during wakefulness and sleep. However, unlike RS1, respiratory control is carried out through a proportional controller that does not take into account the work of breathing done by the subject. It adjusts ${\dot{V}}_{E}$ in function of the brain and arterial partial pressures of *CO*_2_ and *O*_2_ and regulates *f*_*R*_ and *V*_*T*_ through lines predefined in Duffin et al. (2000). Moreover, RS2 considers a multiplicative interaction among peripheral chemoreceptors more than a higher sensitive to hypoxia than hypercapnia (Blain et al., 2010; Cui et al., 2012; Kumar and Prabhakar, 2012), such as it is described in RS1 (see Equations 3, 4). It also does not take into account the control neural performed by the central controller to changes in the subject's metabolic rates during exercise (Williamson, 2010; Duffin, 2014; Guyton, 2015). Furthermore, it is not clear why in RS2 both *PaO*_2_ and *SaO*_2_ were indicated as independent variables since peripheral chemoreceptors are more sensitive to *PaO*_2_ rather than *SaO*_2_ (Kumar and Prabhakar, 2012). Simulations of this model, using exercise stimuli, did not show a proper regulation of blood gases for both transient and steady-state responses. This fact is understandable if it considers that RS2 is a model designed for sleep-related studies and not for another kind of stimuli such as exercise. One of the challenges of this study was to adapt RS2 to simulate exercise.

The third analyzed model, RS3, was proposed in this study to take advantages of completeness and versatility of RS2 and some properties of RS1. This model was proposed integrating key features of RS1 into RS2. In this sense, the proposed model, RS3, provided to RS2 the ability to adjust the ventilation in function of (a) the brain partial pressure of *CO*_2_ and the arterial partial pressure of *CO*_2_ and *O*_2_ and (b) the tissue metabolic demand. Unlike RS2, RS3 controller allows describing the highest sensitive of such chemoreceptors to hypoxia (see second and third terms of Equations 3, 4). Additionally, this model also supplied to RS2 the capacity to adjust breathing pattern considering an efficiency criterion based on minimization of work of breathing through regulation of *f*_*R*_ (see Equation 9). These features enable RS3 to accomplish appropriate transient and stationary responses during exercise. Likewise, the model improvement is not only related to prediction error. RS3 showed transient responses faster than RS2 with a better physiological meaning. This was especially important in the dynamics found for *P*_*aCO*_2__ and *P*_*aO*_2__ (see Figure 4), which were consistent with results published in previous studies (Krogh and Lindhard, 1913; Pearce and Milhorn, 1977; Whipp et al., 1982; Mateika and Duffin, 1995; Turner et al., 1997; Whipp and Ward, 1998; Magosso and Ursino, 2005; Wasserman et al., 2011; Parkes, 2013; Guyton, 2015). On the other hand, although RS3 is more complex and complete than RS1, there is not a higher complexity in RS3 respect to RS2, but there is a substitution of some blocks and equations (from RS1) that allowed getting the improvements mentioned above.

Even though RS3 showed a good regulation of ventilation and blood gases partial pressures and, unlike RS1, it provides information related to cardiac activity such as heart rate, stroke volume, and cardiac output, we are still far to reproduce a real response to this type of stimulus. One of the found handicaps is related to many theories that have been developed so far to describe the underlying mechanism to the cardiorespiratory response during exercise. Particularly, RS3 (like RS1) has a component in its controller that allows it to simulate exercise stimulus from metabolic rate ratio (MRR) and, therefore, from ${\dot{V}}_{CO_{2}}$ and ${\dot{V}}_{O_{2}}$ (see Equations 3, 6). This feature allowed RS3 to reproduce transient responses similar to those reported in the literature, especially at the onset of exercise, and achieve a better performance when experimental data were used. We are aware that this “exercise component” is a straightforward approach to simulate this type of control and additional efforts will be necessary to reproduce detailed changes in ventilation due to, for example, the central command, large vessels chemoreceptors and mechanoreceptors located in exercising muscles. Nonetheless, we consider results obtained here represent one-step beyond simulation of this kind of stimulus because they allowed contrasting the models, taking advantages of their key features, reproducing transient responses more realistic from a physiological point of view and getting a better prediction error.

Another handicap is related to published studies include many variables that influence the subject's response: mode exercise (walking, pedaling), posture, initial conditions, state of the subject (trained, inexperienced, uncomfortable, anxious, anticipating, distracted, tired, etc.), and metabolic rate (Bell, 2006; Fadel, 2013; Duffin, 2014). On the other hand, due to the significant individual variation in the ventilatory responses reported in the literature, a better prediction of real breathing patterns can be achieved only by fitting procedures (i.e., estimation of individual parameters).

Finally, the proposed model RS3 will need a validation in a sample of healthy controlled subjects in different exercise conditions to go in deep in the controller mechanism of the respiratory system during exercise.

## Author contributions

All authors made substantial contributions to the conception and design of the paper. LS: Carried out the model simulations, processed the obtained data, and drafted the review; MM, AH, and RR: Revised it critically for content; All authors approved the final submission of the document and agreed to be accountable for all aspects of the work.

### Conflict of interest statement

The authors declare that the research was conducted in the absence of any commercial or financial relationships that could be construed as a potential conflict of interest.
