# Supplementary material for: An Improved Dynamic Model for the Respiratory Response to Exercise
Source: Front Physiol. 2018 Feb 7;9:69. doi: 10.3389/fphys.2018.00069 (PMC5808356; doi:10.3389/fphys.2018.00069)
Supplement: Supplementary file 1 [file DataSheet1.pdf]

Supplementary Material

An Improved Dynamic Model for the Respiratory Response to Exercise

L.Y. Serna*, M.A. Mañanas, A.M. Hernández, and R.A. Rabinovich.

*** Correspondence:** L.Y. Serna, Biomedical Engineering Research Centre (CREB), Universitat Politècnica de Catalunya, C/ Pau Gargallo 14, 08028, Barcelona, Spain. leidy.yanet.serna@upc.edu.

1 Supplementary Figure 1

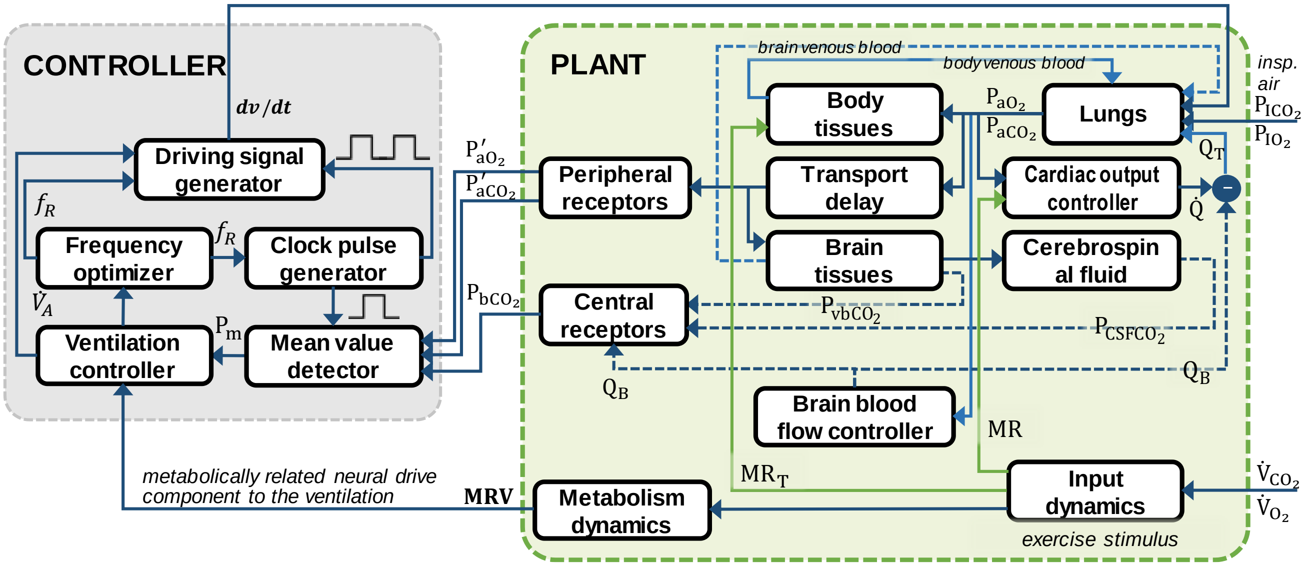

Figure S1. Block diagram of the RS1 model describes in (Fincham and Tehrani, 1983). $PaCO_2$ and PaO_2 denote the partial pressures of CO_2 and O_2 in the arterial blood; P_{bCO_2} and P_{vbCO_2}, the partial pressure of CO_2 in the brain blood and brain venous blood respectively; and P_{CSFCO_2} denotes the partial pressure of CO_2 in the cerebrospinal fluid. The prime (') sign denotes the delayed version of $PaCO_2$ and PaO_2 due to arterial transfer. In the model, mean values of P'_{aCO_2}, P'_{aO_2} and P_{bC}[illegible] (P_m) and a metabolically related neural drive component to the ventilation (MRV) are used by the controller to compute the alveolar ventilation ($\dot{V}_A$) and adjust the respiratory frequency each breathing cycle following the optimization principle set in (Otis et al., 1950).

2 Supplementary Figure 2

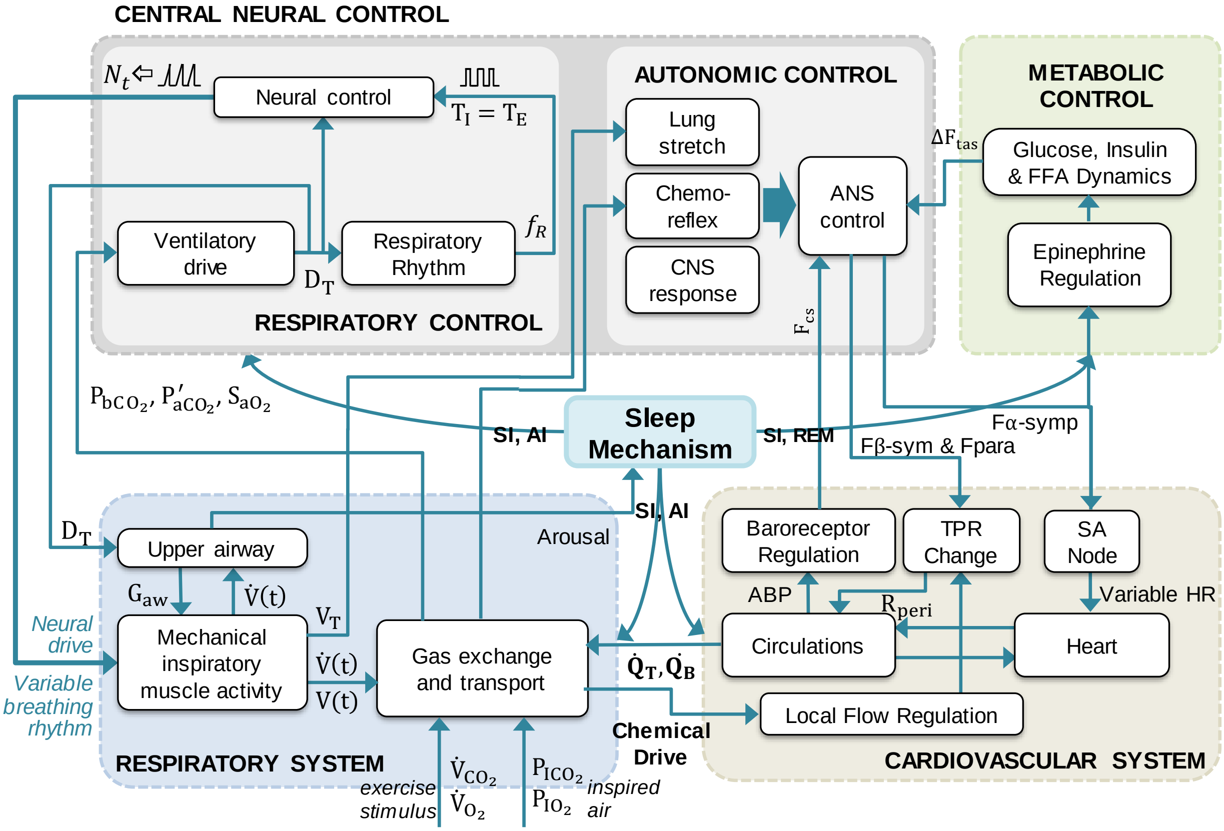

Figure S2. Block diagram of the RS2 model describes in (Cheng et al., 2010; Cheng and Khoo, 2012). $PaCO_2$ and PaO_2 denote the partial pressures of CO_2 and O_2 in the arterial blood; P_{bCO_2} the partial pressure of CO_2 in the brain blood; and SaO_2 denotes the arterial saturation of O_2. In this model, D_T denotes the ventilatory demand and it is adjusted by a proportional controller that computes the respiratory frequency (f_R) following predefined lines in (Duffin et al., 2000).

Acronym

- CNS: Central nervous system
- ANS: Autonomic nervous system
- FFA: Free fat acids
- TPR: Total peripheral resistance

3 Supplementary Table 1

Table S1. Model variables and symbols. Only relevant variables are described below. For more detailed information, consult the following references (Cheng et al., 2010; Fincham and Tehrani, 1983).

Variable	Definition	Value	Units
$\dot{V}_E$	Minute ventilation	--	L/min
f_R	Respiratory frequency	--	breaths/min
V_T	Tidal Volume	--	L
T_I	Inspiratory time	--	s
T_E	Expiratory time	--	s
T_{TOT}	Breathing cycle	--	s
$\dot{V}_A$	Alveolar ventilation	--	L/s
$\dot{V}_{A_{basal}}$	Basal alveolar ventilation (RS1 and RS3)	0.0673	L/s
V_D	Dead space volume	--	L
R_{rs}	Respiratory system resistance (RS1, RS2 and RS3)	8.55	$cmH_2O/L/s$
E_{rs}	Respiratory system elastance (RS1, RS2 and RS3)	3.1	cmH_2O/L
MR_{BCO_2}	Metabolic rate in brain tissues for CO_2	0.000925	L/s
MR_{BO_2}	Metabolic rate in brain tissues for O_2	0.009	L/s
MR_{TCO_2}	Metabolic rate in body tissues for CO_2 (basal rate → $0.20/60$ L/s)[1]	--	L/s
MR_{TO_2}	Metabolic rate in body tissues for O_2 (basal rate → $0.25/60$ L/s) [1]	--	L/s
MRR	Metabolic rate ratio	--	Dimensionless
MRV	Metabolic neural drive (exercise neural component) [2]	--	Dimensionless
τ_3	Exercise metabolic dynamic (RS1 and RS3)	30	s
τ_4	Metabolically derived neural drive (RS1 and RS3)	50	s
$\dot{V}_{O_2}$	Ventilation (consumption) of O_2[1]	--	L/s
$\dot{V}_{CO_2}$	Ventilation (production) of CO_2[1]	--	L/s
RTT	Metabolic input function to issue	--	L/s
Q_B	Blood flow rate in brain	--	L/s
Q_T	Blood flow rate in tissues	--	L/s
$PetCO_2$	End tidal partial pressure of CO_2 [2]	--	mmHg
$PaCO_2$	Partial pressure of CO_2 in the arterial blood [2]	--	mmHg
PaO_2	Partial pressure of O_2 in the arterial blood [2]	--	mmHg
$PbCO_2$	Partial pressure of CO_2 in the brain blood [2]	--	mmHg
$PbvCO_2$	Partial pressure of CO_2 in venous brain blood	--	mmHg
P_{CSFCO_2}	Partial pressure of CO_2 in the cerebrospinal fluid	--	mmHg
D_T	Ventilatory demand (RS2)	--	L/s
I_C	Central chemoreceptor activation (RS2)	45	Dimensionless
I_{pCO_2}	Peripheral chemoreceptor threshold for CO_2 (RS2)	38	Dimensionless
I_{pO_2}	Peripheral chemoreceptor threshold for O_2 (RS2)	102.4	Dimensionless
G_c	Gain for central chemical drive (RS2)	0.075	Dimensionless
G_p	Gain for peripheral chemical drive (RS2)	0.0063	Dimensionless
F_b	Basal breathing frequency (RS2)	12.5	Breath/min
V_b	Basal ventilation (RS2)	6.7	L/min
T_D	Chemoreflex drive threshold (RS2)	1539	mL

Table S1. Continuation

Variable	Definition	Value	Units
T_P	Chemoreflex drive threshold (RS2)	2879	mL
S_{1F}	Scaling factor (RS2)	0.00518	Dimensionless
S_{2F}	Scaling factor (RS2)	0.0105	Dimensionless
N	Neuromuscular drive (RS2) [3]	Variable	Dimensionless
SI	Sleep state index (It provides an indication of whether the model is "awake" (SI=0) or "asleep" (SI=1). [4]	0	Dimensionless
AI	Arousal index [2]	1	Dimensionless
REM	Rapid eye movement (1for REM phase, 0 for no-REM fase) [4]	0	Dimensionless
HR	Heart rate	--	Beats/min
ABP	Arterial blood pressure	--	mmHg
VC	Vital capacity (RS2 and RS3)	5	L
RC	Muscle constant time	0.060	s

[1] Variables $\dot{\mathbf{V}}_{\mathbf{CO_2}}$ and $\dot{\mathbf{V}}_{\mathbf{O_2}}$ were considered equivalent to $\mathbf{MR_T}$ for $\mathbf{CO_2}$ and $\mathbf{O_2}$, respectively . Therefore, these variables were used to simulate exercise.
[2] Ventilation excitatory variables
[3] Muscular activity excitatory variable
[4] Ventilation inhibitory variables
